# IndiaScene365: a transfer learning dataset for Indian scene understanding in diverse weather condition

**DOI:** 10.3389/frai.2025.1669512

**Published:** 2026-01-09

**Authors:** Deepa Mane, Sandhya Arora, Sachin Shelke

**Affiliations:** 1 Department of Computer Engineering, Smt. Kashibai Navale College of Engineering, SPPU, Pune, India; 2 Department of Computer Engineering, Cummins College of Engineering for Women, SPPU, Pune, India; 3 Department of Information Technology, Pune Institute of Computer Technology, SPPU, Pune, India

**Keywords:** deep learning, pre-trained models, scene understanding, transfer learning, domain adaptation

## 1 Introduction

Autonomous driving systems are inherently reliant on strong object detection to effectively sense and traverse intricate real-world scenes. While deep learning, and more specifically convolutional neural networks (CNNs), have powered much of the growth in this area, most public benchmarks like KITTI (Varma et al., 2019), BDD10K and Cityscape (Maddern et al., 2017) draw from strongly structured, rule-abiding traffic scenarios found in Western nations. These settings are quite far from those in countries such as India, where unstructured traffic, uneven infrastructure, and extreme environmental fluctuations prevail. Because diversity is so high in India, it provides an ideal setting to create generalizable perception models able to deal with uncertainty and real-world complexity. Some Indian datasets like the Indian Driving Dataset (IDD) (Varma et al., 2019; Paranjape and Naik, 2022), ITD (Agarwal et al., 2024), DATS (Srinath et al., 2020), NITCAD (Krizhevsky et al., 2017), and the latest multimodal TIAND dataset (Yang et al., 2019) Sdac (Gong et al., 2024) have been proposed to overcome these challenges. Although all of them provide useful insights, they generally concentrate on individual tasks (e.g., semantic segmentation), few object classes, or mainly urban environments, leaving a gap for an extensive, large-scale dataset focused on heterogeneous object detection in unstructured Indian traffic scenes. IndiaScene365: A transfer Learning dataset for Indian Scene Understanding in diverse weather conditions comprises images from traffic environments that truly represents Indian roads. Unlike other standard available datasets that feature well-organized traffic scenes from various global locations, this collection includes vehicle types such as motorcycles, auto-rickshaws, and animal-drawn carts, which are characteristic of Indian road conditions but absent from worldwide datasets. Researchers interested in using this dataset can be downloaded directly from Mendeley data at https://data.mendeley.com/datasets/pwffhg6nhz/1 or contact the author directly (Mane, 2022).

## 2 Background

In recent years, numerous datasets for advanced driver assistance systems and autonomous car driving have emerged, primarily focusing on structured and well-defined driving environment. These typically feature well-defined lanes, a limited number of clearly categorizable objects, minimum difference between object and background and strict adherence to traffic rules. The Oxford Robotcar dataset pioneered large-scale real-world data collection (Maddern et al., 2017), including significant representation of challenging visual traffic scenes such as low illumination, rain, nighttime and foggy but it might not cover extreme weather conditions like snow, heavy rain, or fog in sufficient detail (Yin et al., 2024). This limits the ability to train models that can generalize well to such conditions. Foggy Zurich (Sakaridis et al., 2018) and Foggy Cityscapes (Paranjape and Naik, 2022) introduced synthetic fog images by applying masks to original images. Synthetic rain datasets (Zheng and Yoo, 2025) include Rain1400, RainyCityscapes Rain100H, and Rain12. Datasets like, BDD100K, ACDC (Dokania et al., 2023), and IDD (Varma et al., 2019; Agarwal et al., 2024), contain authentic images collected from various adverse conditions (Yin et al., 2024), including fog, rain, snow, and nighttime scenarios (Srinath et al., 2020; Krizhevsky et al., 2017). However, most of these datasets are prepared based on structured environments and have inadequate annotations for unstructured scenarios. Table 1 Comparison of major Indian and global object detection datasets shows the comparative analysis of all the other datasets we considered and novelty of our dataset. Below is the Label Hierarchy that we have used in our dataset. The dataset uses a hierarchical labeling framework based on a parent-child relationship to represent the semantic relationship between traffic entities on a broad scale and a finer-grained scale. Classes are organized into a wider parent class, which is organized into a child sub-class. For example, Vehicles → Car, Bus, Truck, Auto-rickshaw. This hierarchy supports flexible training, allowing algorithms to be trained using either flat labels for classification/segmentation for a single layer or hierarchical supervision for better generalization across similar classes.

**Figure:**
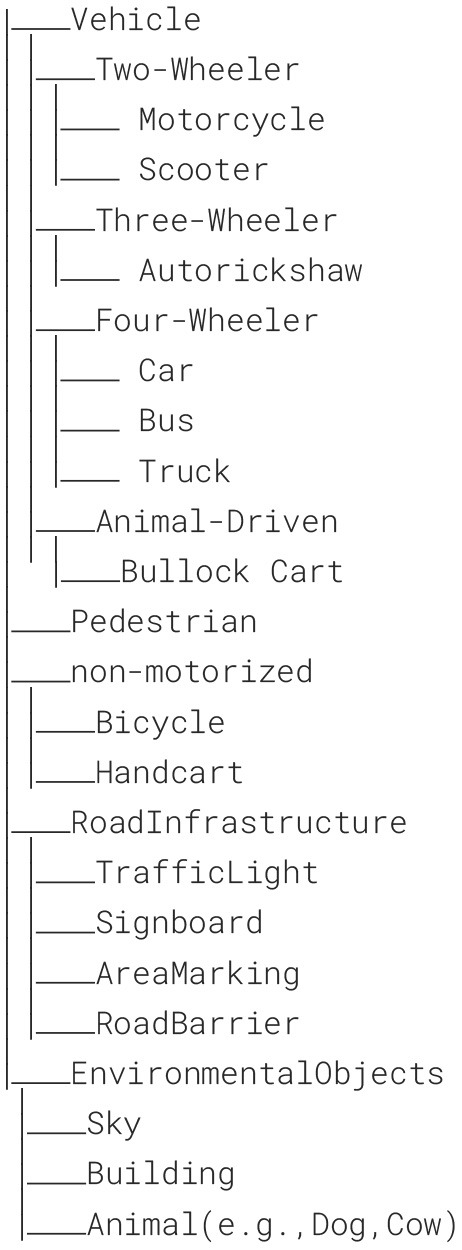


**Table 1:** Comparison of major Indian and global object detection datasets.

**Datasets**	**Region**	**Classes**	**ARW**	**LCV**	**Animal cart**	**Rain/fog/sunny**	**Annotation type**
Kitti	Germany	28	No	No	No	No	MOT
BDD100K	USA	10	No	No	No	Yes	Detection/Segm./tracking
Cityscape	Germany	30	No	No	No	No	Semantic + instance segmentation
IDD	India	34	Yes	Yes	Yes	No	3D object detection
ACDC	Europe	19	No	No	No	Yes	Semantic + instance segmentation
Indiascene365	India	34	Yes	Yes	Yes	Yes	Object detection

### 2.1 Dataset overview

Indiascene365 is a dataset comprising over 3,000 images captured using Android smartphone (One Plus Nord CE) equipped with 50 MP super cameras and unplug car mounted stereo front and back camera. These devices produce images of quality comparable to professional cameras. The increasing prevalence of mobile phones, which are preferred over bulky cameras, makes this mobile-captured dataset more practical and representative. The images were taken across various locations in Maharashtra, India, encompassing different road types and traffic conditions as detailed in Table 2 Details of Data collection Sources. The dataset includes photos taken during the day and evening, across all seasons. The diverse locations range from bustling city streets and 6-lane national highways with varying traffic densities to rural roads with animal presence (Li et al., 2023). Table 2 showcases depicting national highways, rural roads, construction-affected highways, crowded urban streets, mountain roads with wildlife, traffic at different times of day, highways with large vehicles, and animals on roadways. The mountain roads feature sharp turns bordered by dense forests or steep cliffs and deep valleys.

**Table 2:** Details of data collection sources.

**Location**	**Types of roads**	**Season**	**Structured/unstructured**
Pune-Bangalore National highway-48	A 6 lane Spanning 2,807 kilometers, the National Highway of India extends across seven states within the country.	Summer	Structured
In and around Pune, Rajiv Gandhi Udyaan	Khashaba Jadhav path Lane no. 6		Mixed structure and unstructured
Mumbai Goa National Highway (NH 66)	4 lane spanning approximately 1,608 km along India's western shoreline, a bustling National Highway with 4 lanes extends in a generally north-south direction	Rainy	Structured
In and around Pune (2nd-largest city in the state of Maharashtra, India)	Jalvaayu marg ambey valler rd. Lonavala		Mixed structure and unstructured
The Mumbai–Pune Expressway is India's first 6-lane wide concrete, access-controlled tolled expressway.	The route extends approximately 94.5 km, linking Raigad-Navi Mumbai with Maharashtra's state capital and India's financial hub.	Winter	Structured
(Lonavala city in the state of Maharashtra, India)	NH 48 Tiger valley khandala ghat Lonavala		Mixed somewhat structured and unstructured

### 2.2 Data classes

The label set used in dataset preparation is identical to that in IDD (Varma et al., 2019; Agarwal et al., 2024) The hierarchy in the category of class labels adds a higher degree of complexity (Srinath et al., 2020) to our dataset compared to existing datasets like Cityscapes (Yang et al., 2019), and even when compared to adverse weather datasets such as Foggy Cityscapes (Geiger et al., 2013; Baker et al., 2011). A team of highly skilled annotators was employed to label the dataset. The labelling process for each weather condition images typically take 2–3 h, including initial annotation and review process. To ensure annotation accuracy, multiple revisions were taken, along with verifying annotations against previously defined classes and conducting a final validation by expert annotators. Indiascene365 is split into three sets corresponding to the examined conditions. We took around 1,000 lowlight and daylight, around 1,000 rain and 1,000 foggy images from selected recordings for detailed annotation process, resulting in 3,000 images depicting adverse conditions. The selection criteria focused on maximizing scene complexity and diversity (Sakaridis et al., 2021). To ensure robust evaluations, we implemented a comprehensive train-test split method with defined parameters. All images from a single drive sequence were allocated entirely to either the train or test set, guaranteeing that test data will remain novel. The test set for each weather condition included drive sequences comprising 18% to 20% of total scenes, ensuring a representative sample of various driving scenarios. In the test set, we kept the mean number of frames per drive sequence between 90% and 120% of the dataset's overall average. To ensure fairness across classes, we limited the average number of instances per image in the test set to between 80% and 120% of the entire dataset's average. To guarantee accurate pixel-level annotations, test images were required to have an average pixel ratio ranging from 0.5 to 1.4 for at least 18 classes, and from 0.4 to 1.5 for a minimum of 24 classes.

### 2.3 Data formats and file structure

**Figure 1:**
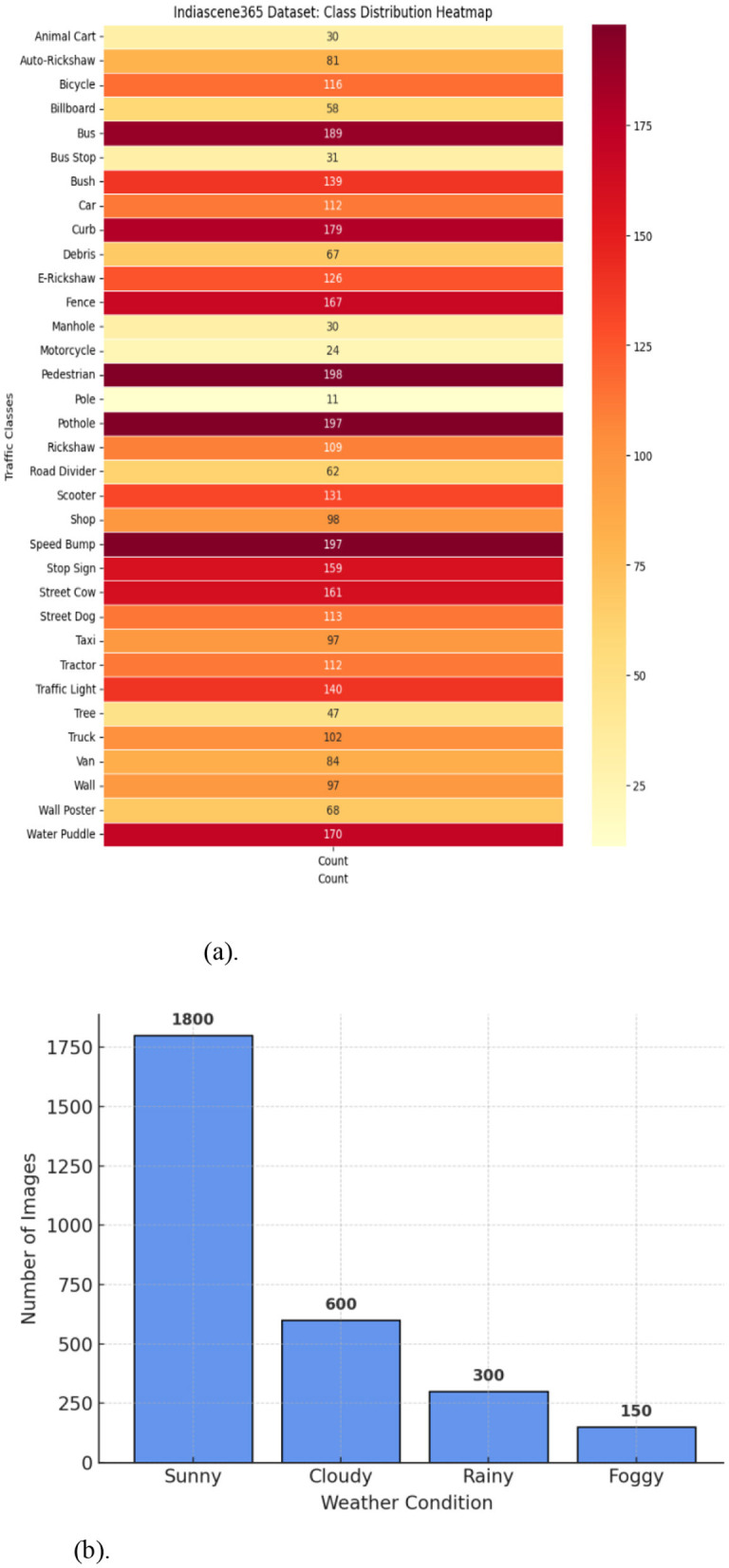
**(a)** Class distribution heatmap. **(b)** Weather condition distribution vs. number of images.

**Figure 2:**
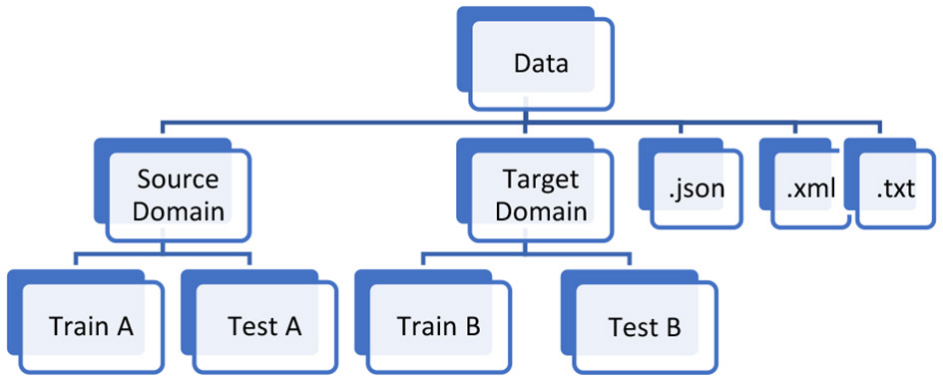
Data folder layout.

**Figure 3:**
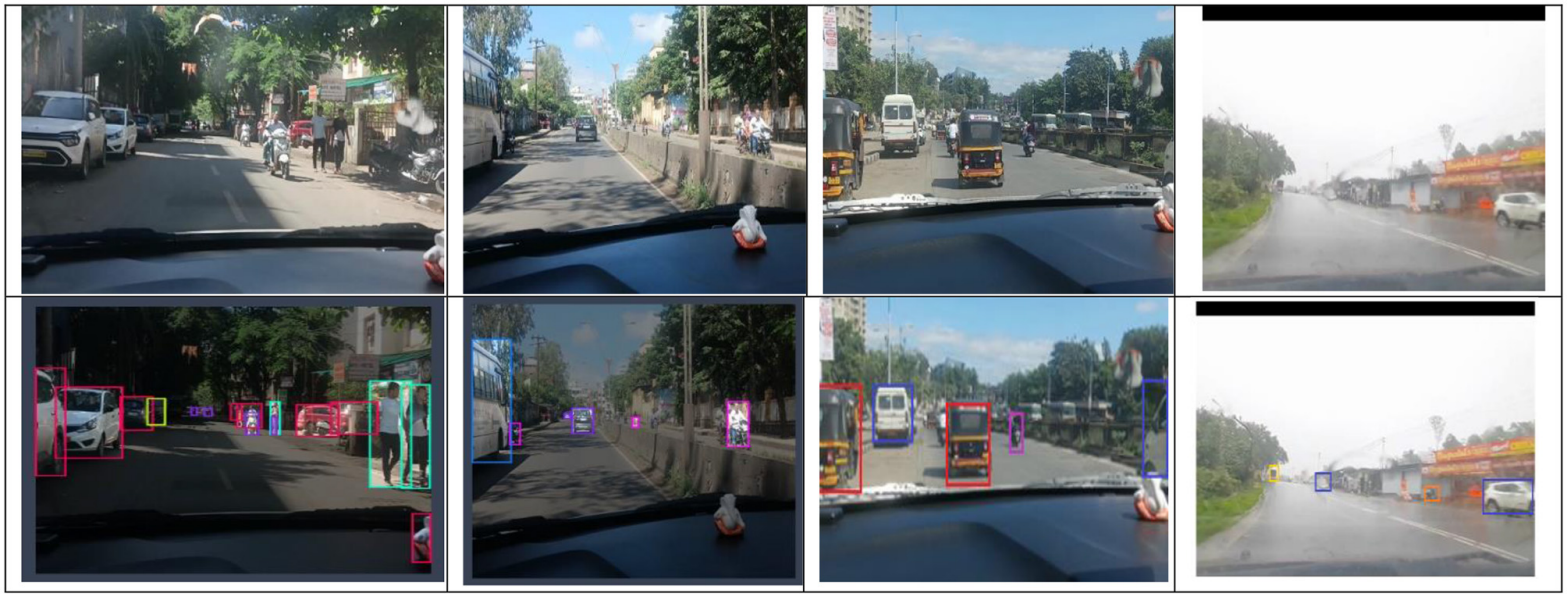
Sample Images of diverse road conditions at different seasons on left side and their respective annotated images with bounding boxes.

The dataset comprises diverse road scenes, including national highways, country roads, construction zones, busy city streets, and mountain passes. It showcases traffic patterns at various times of day, as well as different weather conditions such as rain and fog. The collection features highways with large vehicles and instances of animals on roads. Mountain routes are characterized by sharp turns, with dense forests on one side and steep peaks or valleys on the other. In rural areas, trees line both sides of the roads. The aim of forming this dataset is to capture images representing various outdoor scenes throughout the city, encompassing different weather scenarios and times of day. High quality videos and images were collected using smartphone cameras. Afterward frames were extracted from video streams as images using VLC media player at 30 fps. Indiascene365 classifies objects into 34 distinct categories, organized into broader groups, as show Figure 1. A thorough dataset should include data descriptions, or annotations, alongside the images. Data annotation involves defining areas or objects within an image and creating text descriptions for them. Figure 2 represents the data structure of the data in the repository. The open-source Roboflow tool^1^ was used to annotate images in the proposed dataset. After completing image annotation and saving, an XML file is generated for each image and needs to maintain the different folders for different weather conditions as well as for XML and annotations files. These XML files have details about each labeled object in the image and its coordinates in the images, specifying the annotation type as “bounding box”. Additionally, .txt and .json files are generated with reference each image and stored in the respective folders. The dataset comprises diverse road scenes, including national highways, country roads, construction zones, busy city streets, and mountain passes. It showcases traffic patterns at various times of day, as well as different weather conditions such as rain and fog. The collection features highways with large vehicles and instances of animals on roads. Mountain routes are characterized by sharp turns, with dense forests on oneside and steep peaks or valleys on the other. In rural areas, trees line both sides of the roads. Figure 3 shows sample Images of diverse road conditions at different seasons and their respective annotated images with bounding boxes.” The aim of forming this dataset is to capture image representing various outdoor scenes throughout the city, encompassing different weather scenarios and times of day. High quality videos and images were collected using smartphone cameras. Afterward frames were extracted from video streams as images using VLC media player at 30 fps.

## 3 Materials and methods

**Figure 4:**
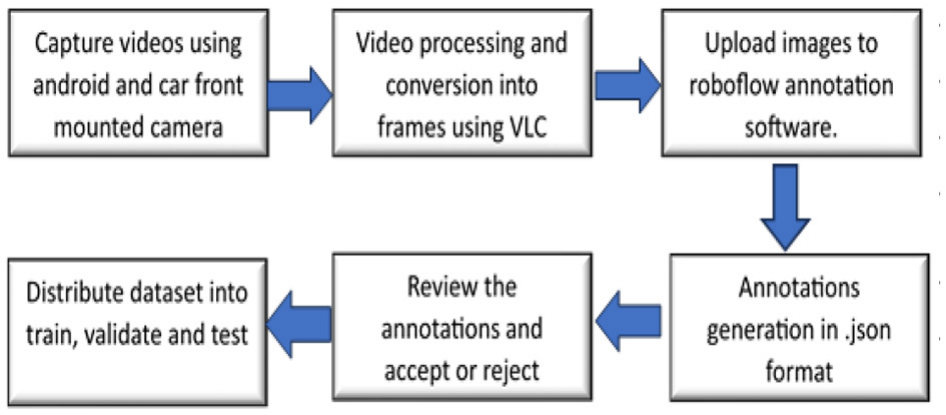
Dataset generation process.

Dataset Creation Process and Methodology: Figure 4 illustrate the steps involved in generating the dataset. Images were collected from various roadways within Pune city and its surrounding areas in Maharashtra, India. The dataset was compiled by the authors and team members using a standard Android smartphone camera and car mounted stereo front and back camera over a 1-year period. Consequently, the image count is lower compared to other datasets. However, image collection will be used for semantic segmentation soon, and the dataset will be revised periodically.

### 3.1 Experiments

#### 3.1.1 . Experiment 1

A Faster RCNN-based object detection model was pre-trained on global datasets (Cityscapes, BDD100K) and fine-tuned on IndiaScene365 to evaluate cross-domain adaptation performance.

#### 3.1.2 Experiment 2

A YOLOv11-based object detection model was pre-trained on global datasets (Cityscapes, BDD100K) and fine-tuned on IndiaScene365 to evaluate cross-domain adaptation performance.

### 3.2 Classification results

**Figure 5:**
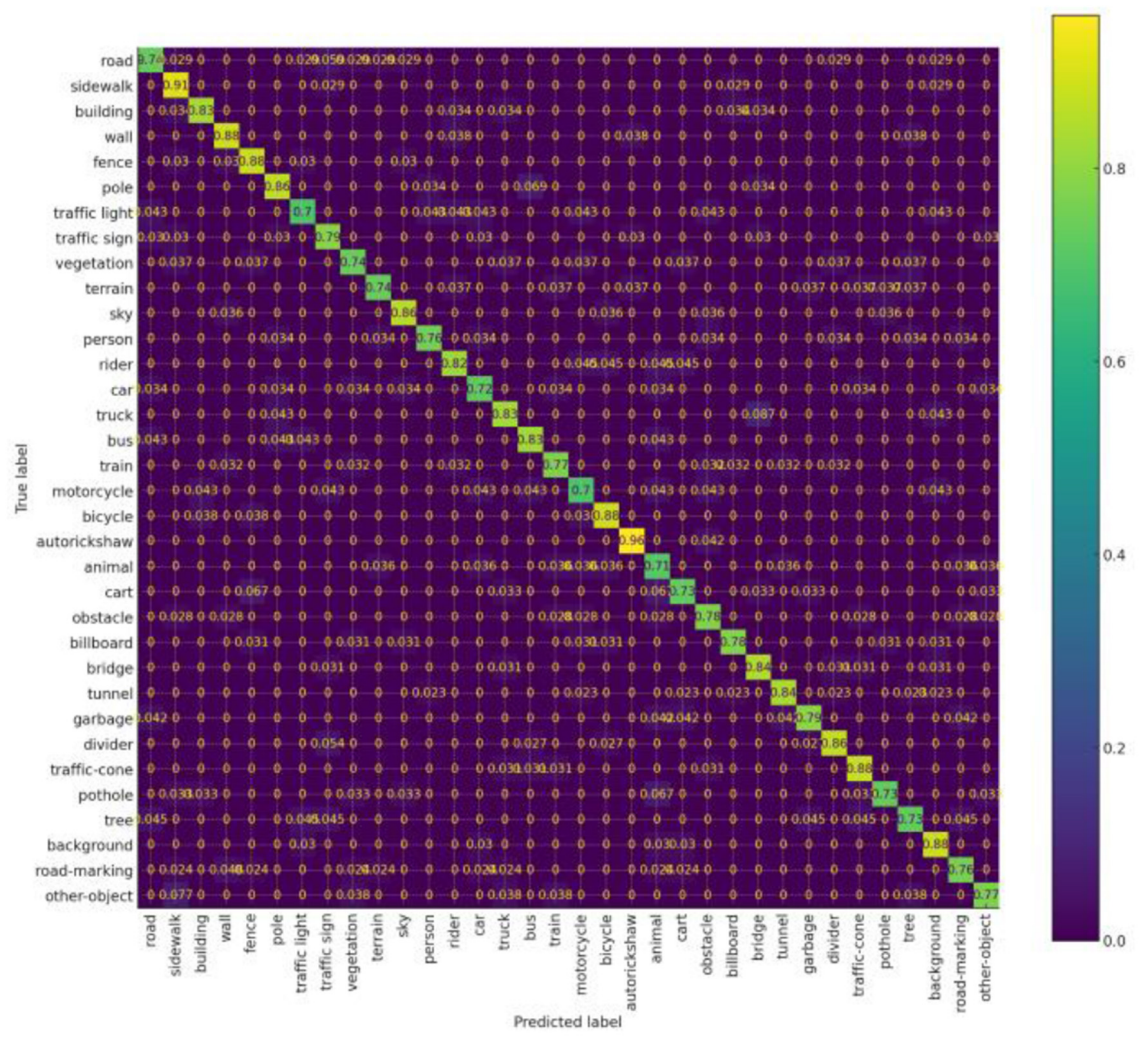
Confusion matrix.

To evaluate the effectiveness of our dataset in developing accurate deep learning models for classifying the objects in scene understanding we evaluated with object detectors like Faster RCNN and a Yolov11. More details about the methodology and configuration of the experiments, are described in 3.1. Here we present the results obtained for both the experiments. Table 3 summarizes the results and Figure 5 Shows the confusion matrix for the second experiment.

**Table 3:** Classification results.

**Experiment**	**Method**	**Accuracy**
1	Faster RCNN	87.2
2	yolov11	94.7

### 3.3 Data quality assurance

To confirm the reliability and diversity of the dataset, we conducted an image quality and redundancy assessment. Quality was assessed with objective metrics, including BRISQUE (Blind/Referenceless Image Spatial Quality Evaluator) for perceptual quality, variance of the Laplacian level as a measure of sharpness, and mean brightness and contrast measures to evaluate light consistency. Frames that exhibited extremely low levels of sharpness or presented abnormal light conditions were either tagged for manual review or discarded. Redundancy arising from sequential video input required an approach to identify near-duplicate frames through perceptual hash (pHash)-based analysis of visual similarity. Selecting a cutoff of 95% similarity, we disseminated frames that responded to the threshold and were visually distinct. In this way, we took slight actions to reduce redundancy and maintain level of representational diversity across different weather and scenes, limiting any implications of redundancy or similarity for the representational sample.

## 4 Limitations and future scope

This transfer learning dataset is obtained from sparse domains, which could limit its generalizability and versatility in low-resource settings. Despite the variety provided by Indiascene365, there are still some limitations, providing openings for future improvement:

Geographically this dataset largely reflects western Indian regions and fails to completely cover the varied terrains, traffic conditions, and signage observed in other parts of India—e.g., hilly regions or densely populated urban areas in the north and northeast. Safety-critical but less frequent classes (e.g., ambulances, police cars) are not well-represented, which results in difficulties for robust model training on long-tail classes. Indiascene365 has just RGB images at present. Adding other modalities like LiDAR would further improve perception performance in the presence of adverse weather and complex road geometry.

Limited Annotation Depth: The data offers only 2D bounding box annotations. Including semantic segmentation masks or 3D bounding boxes (e.g., point-level LiDAR) would make it more useful for more general autonomous vehicle perception tasks.

looking forward Indiascene365 as a starting point benchmark to propel research in strong, multimodal perception for unstructured roadway environments.

## Data Availability

The datasets presented in this study can be found in online repositories. The names of the repository/repositories and accession number(s) can be found in the article/supplementary material.

## Footnotes

1 https://docs.roboflow.com/annotate/annotation-tools.

## References

[B1] Agarwal A. Thombre A. Kedia K. Ghosh I. (2024). “Itd: Indian traffic dataset for intelligent transportation systems,” in 2024 16th International Conference on COMmunication Systems and NETworkS (COMSNETS) (Bengaluru: IEEE), 842–850. doi: 10.1109/COMSNETS59351.2024.10427394

[B2] Baker S. Scharstein D. Lewis J. P. Roth S. Black M. J. Szeliski R. (2011). A database and evaluation methodology for optical flow. Int. J. Comput. Vis. 92, 1–31. doi: 10.1007/s11263-010-0390-2

[B3] Dokania S. Hafez A. H. A. Subramanian A. Chandraker M. Jawahar C. V. (2023). “Idd-3d: Indian driving dataset for 3d unstructured road scenes,” in Proceedings of the IEEE/CVF Winter Conference on Applications of Computer Vision (WACV), 4482–4491. Available online at: https://cdn.iiit.ac.in/cdn/cvit.iiit.ac.in/images/ConferencePapers/2023/IDD-3D.pdf (Accessed October 23, 2025).

[B4] Geiger A. Lenz P. Stiller C. Urtasun R. (2013). Vision meets robotics: the kitti dataset. Int. J. Robot. Res. 32, 1231–1237. doi: 10.1177/0278364913491297

[B5] Gong L. Zhang Y. Xia Y. Zhang Y. Ji J. (2024). Sdac: a multimodal synthetic dataset for anomaly and corner case detection in autonomous driving. Proc. AAAI Conf. Artif. Intell. 38, 1914–1922. doi: 10.1609/aaai.v38i3.27961

[B6] Krizhevsky A. Sutskever I. Hinton G E. (2017). ImageNet classification with deep convolutional neural networks, Commun. ACM 60, 84–90. doi: 10.1145/3065386

[B7] Li J. Kaur S. S. Do M. N. (2023). “Domain adaptive object detection for autonomous driving under foggy weather,” in Proc. IEEE Winter Conf. on Applications of Computer Vision (WACV) (Waikoloa, HI: IEEE), 4332–4341. doi: 10.1109/WACV56688.2023.00068

[B8] Maddern W. Pascoe G. Linegar C. Newman P. (2017). 1 year, 1000 km: the oxford robotcar dataset. Int. J. Robot. Res. 36, 3–15. doi: 10.1177/0278364916679498

[B9] Mane D. (2022). Indiascene365, Mendeley Data V2 (Mendeley: Elsevier).

[B10] Paranjape B. A. Naik A. A. (2022). Dats_2022: a versatile indian dataset for object detection in unstructured traffic conditions. Data Brief 43:108470. doi: 10.1016/j.dib.2022.108470 35898859 PMC9309657

[B11] Sakaridis C. Dai D. Van Gool L. (2018). Semantic foggy scene understanding with synthetic data. Int. J. Comput. Vis. 126, 973–992. doi: 10.1007/s11263-018-1072-8

[B12] Sakaridis C. Wang H. Li K. Zurbrugg R. Jadon A. Abbeloos W. . (2021). ACDC: The Adverse Conditions Dataset with Correspondences for Robust Semantic Driving Scene Perception (IEEE). doi: 10.1109/ICCV48922.2021.01059 41237037

[B13] Srinath N. G. S. S. Joseph A. Z. Umamaheswaran S. Priyanka C. L. Nair M. Sankaran P. (2020). NITCAD-Developing an object detection, classification and stereo vision dataset for autonomous navigation in Indian roads. Proc. Comput. Sci. 171, 207–216. doi: 10.1016/j.procs.2020.04.022

[B14] Varma G. Subramanian A. Namboodiri A. Chandraker M. Jawahar C. V. (2019). “IDD: a dataset for exploring problems of autonomous navigation in unconstrained environments,” in 2019 IEEE Winter Conference on Applications of Computer Vision (WACV) (IIT Hyderabad, IEEE), 1743–1751. doi: 10.1109/WACV.2019.00190

[B15] Yang W. Tan R. T. Feng J. Guo Z. Yan S. Liu J. (2019). Joint rain detection and removal from a single image with contextualized deep networks. IEEE Trans. Pattern Anal. Mach. Intell. 42, 1377–1393. doi: 10.1109/TPAMI.2019.2895793 30703011

[B16] Yin H. Wang P. Liu B. Yan J. (2024). An uncertainty-aware domain adaptive semantic segmentation framework. Auton. Intell. Syst. 4:15. doi: 10.1007/s43684-024-00070-0

[B17] Zheng X. Yoo C. D. (2025). DriVLM: domain adaptation of vision-language models in autonomous driving. arXiv preprint arXiv:2501.05081. doi: 10.48550/arXiv.2501.05081

